# Clinical and Functional Outcomes of Patients Receiving Cerebral Reperfusion Therapy: A Stroke Databank Study in Brazil

**DOI:** 10.3389/fsurg.2022.799485

**Published:** 2022-02-25

**Authors:** Natalia Eduarda Furlan, Gustavo José Luvizutto, Pedro Tadao Hamamoto Filho, Silmeia Garcia Zanati Bazan, Gabriel Pinheiro Modolo, Natalia Cristina Ferreira, Luana Aparecida Miranda, Juli Thomaz de Souza, Fernanda Cristina Winckler, Edison Iglesias de Oliveira Vidal, Carlos Clayton Macedo de Freitas, Luis Cuadrado Martin, Rodrigo Bazan

**Affiliations:** ^1^Department of Neurology, Psychology and Psychiatry, Botucatu Medical School (UNESP), Botucatu, Brazil; ^2^Department of Applied Physical Therapy, Federal University of Triângulo Mineiro, Uberaba, Brazil; ^3^Department of Internal Medicine, Botucatu Medical School (UNESP), Botucatu, Brazil

**Keywords:** stroke, cerebral reperfusion, mortality rate, disability, endovascular therapy stroke, endovascular therapy

## Abstract

**Objectives:**

Cerebral reperfusion therapy is recommended for the treatment of acute ischemic stroke. However, the outcomes of patients receiving this therapy in middle- and low-income countries should be better defined. This study aimed to evaluate the clinical and functional outcomes of cerebral reperfusion therapy in patients with ischemic stroke.

**Materials and Methods:**

This retrospective study included patients with ischemic stroke treated with cerebral reperfusion therapy, including intravenous thrombolysis (IVT), mechanical thrombectomy (MT), and IVT with MT. The primary outcomes were death and disability, assessed using the modified Rankin scale (mRS), and stroke severity, assessed using the National Institutes of Health Stroke Scale (NIHSS), after intervention and 90 days after ictus. The association between the type of treatment and the primary outcome was assessed using binary logistic regression after adjusting for confounding variables. Furthermore, receiver operating characteristic (ROC) curves were generated to identify the cutoff point of the NIHSS score that could best discriminate the mRS score in all types of treatments.

**Results:**

Patients (*n* = 291) underwent IVT only (*n* = 241), MT (*n* = 21), or IVT with MT (*n* = 29). In the IVT with MT group, the incidence of death within 90 days increased by five times (OR, 5.192; 95% CI, 2.069–13.027; *p* = 0.000), prevalence of disability increased by three times (OR, 3.530; 95% CI, 1.376–9.055; *p* = 0.009) and NIHSS score increased after IVT (from 14.4 ± 6.85 to 17.8 ± 6.36; *p* = 0.045). There was no significant difference between the initial NIHSS score and that after MT (*p* = 0.989). Patients' NIHSS score that increased or decreased by 2.5 points had a sensitivity of 0.74 and specificity of 0.65, indicating severe disability or death in these patients.

**Conclusion:**

Altogether, a 2.5-point variation in NIHSS score after reperfusion is an indicator of worse outcomes. In our particular context, patients receiving the combination of IVT and MT had inferior results, which probably reflects challenges to optimize MT in LMIC.

## Introduction

Cerebral reperfusion therapy is indicated for ischemic strokes, which account for 80% of all strokes (1). However, interventions should be administered at an optimal time to minimize damage to the central nervous system and increase the chance of better outcomes (2, 3). Early recanalization is possibly the main factor for favorable outcomes after ischemic stroke; rapid and effective recanalization increases the chance of better clinical and neurological outcomes (4).

Several studies have shown the benefits of thrombolysis using tissue-type plasminogen activator (t-PA) in acute stroke (5–11). Currently, the advantages of combined therapy with intravenous (IV) t-PA with other modalities of cerebral reperfusion, such as intra-arterial recanalization, are being evaluated. This type of combination strategy has shown good long-term prognosis in previous studies (12, 13). In addition, there has been increasing evidence of the effectiveness of mechanical thrombectomy (MT), which is an emergency revascularization technique (14, 15). The therapeutic window of MT can be extended to up to 8 h after the onset of stroke symptoms in conjunction with standard care, resulting in better functional outcomes at 90 days after stroke than standard care alone (16).

Thus, cerebral reperfusion therapy is highly recommended for the treatment of the acute phase of stroke. However, the benefits of this therapy are poorly understood in low- and middle-income countries (LMICs) due to few patients receiving cerebral reperfusion therapy in these countries. A meta-analysis reported the rates of t-PA use as 19 and 33% in LMICs and upper middle-income countries, respectively, compared to 50% in high-income countries (17). In Latin America, thrombolysis is used in <1% of the population, despite being available throughout the region (18, 19). It is estimated that 10% of patients with acute ischemic stroke have proximal large artery occlusion in the anterior circulation and present symptoms early enough to qualify for MT within 6 h (20). Furthermore, approximately 9% of patients who present with symptoms between the 6- and 24-h time window may qualify for MT (21).

A critical limitation of cerebral reperfusion therapy is the need for controlled settings, which is an important inclusion criterion for studies that assess this therapy. This has fostered an important gap in knowledge. Adequate integration with prehospital services, availability of an interventional neuroradiology team, and the presence of a comprehensive stroke unit or neurointensive care unit before and after the procedure are needed to successfully execute cerebral reperfusion therapy. In view of these difficulties, both public and private hospitals must periodically analyze the treatment quality indicators of cerebral reperfusion therapy to ensure continued improvement in health services. This study aims to evaluate the clinical and functional outcomes of cerebral reperfusion therapy during hospitalization and 3 months after therapy in patients with ischemic stroke in Brazil.

## Materials and Methods

### Study Design, Setting, and Participants

This was a retrospective Brazilian cohort study. All patients were treated in the acute phase of stroke at a comprehensive stroke unit center at the Botucatu Medical School. After hospitalization, patients were followed up for 90 days at the outpatient clinic between June 2012 and December 2020. Data on all patients were collected from the semi-automatic stroke databank, which was developed by our institution. The databank is integrated with the electronic medical records system to extract clinical data on patients admitted to the stroke unit and identify the main indicators of quality of care using artificial intelligence (22).

### Eligibility Criteria

The inclusion criteria were as follows: patients diagnosed with stroke using clinical and neuroimaging examinations, including computed tomography (CT), angiotomography, and/or magnetic resonance imaging (MRI) and had performed IV thrombolysis (IVT) alone, MT alone or IVT with MT. The IVT was indicated for strokes (ranging for minor to severe stroke, including large vessel occlusions), within 4.5 h of symptom onset, NIHSS > 4, except aphasia; The IVT is also offered as the first option to large vessel occlusions within 4, 5 h of stroke symptoms. MT alone was indicated for stroke between 4.5 and 8 h from the onset of symptoms, NIHSS > 8 and ideally AngioCT demonstrating M1 occlusion and/or associated lesion in internal carotid artery; IVT with MT was indicated for stroke within 4.5 h of symptom onset and maintained NHISS > 8 (until 8 h of symptom onset) and AngioCT demonstrating large vessel occlusion. Participants were excluded if they presented with other neurological diseases, hemorrhagic stroke confirmed by CT or MRI scan, or stroke mimics.

### Data Charting Process

A standardized data extraction form created by authors was used and the following details were recorded from each patient. Two calibrated physicians extracted data from the included patients. All variables (confounding factors and outcomes) were extracted by stroke data bank of Botucatu Medical School. The database on monthly audit by the stroke unit coordinator.

The variable of interest in the study was the type of treatment received by the patient, including IV thrombolysis (IVT) alone, MT alone, and IVT with MT. Patients were treated with a standard IV dose (0.9 mg/kg) and maximum total dose (90 mg) of rtPA, alteplase (Actilyse®), and 10% bolus (maximum bolus dose of 9 mg and infusion of the remainder of the total dose over 60 min) (23). MT was performed using the Solitaire flow restoration stent retriever or the Penumbra aspiration system (24). Either device or both devices were chosen according to the discretion of the intervening neuroradiologist.

The primary outcomes were death and disability, assessed using the modified Rankin scale (mRS) (25), and stroke severity, assessed using the National Institutes of Health Stroke Scale Score (NIHSS) (25), after intervention and at 90 days after ictus. The possible confounding variables evaluated were age, sex, ethnicity, history of diabetes and hypertension, smoking, symptom-to-door time, door-to-treatment time, blood pressure at hospital admission, serum creatinine level, NIHSS score at admission and after thrombolysis, Alberta Stroke Program Early CT Score (ASPECTS) (26), symptomatic hemorrhagic transformation according to the European Cooperative Acute Stroke Study (ECASS) criteria (27), and Thrombolysis in Cerebral Infarction (TICI) score, with the aim of grading the technical results of recanalizing therapies in acute ischemic stroke. Successful recanalization was defined as TICI of 2b (50–99% reperfusion) or 3 (complete reperfusion), decompression craniectomy, death on admission, and death and disability within 90 days of stroke.

### Statistical Methods

Non-categorical data are presented as mean ± standard deviation. The normality of continuous variables was assessed by Kolmogorov-Smirnov test. The patients were divided into three groups according to the type of treatment received (isolated IVT, isolated MT, and IVT with MT). Non-categorical data were compared between groups using analysis of variance (parametric distribution variables) or the Kruskal–Wallis test (non-parametric distribution variables), and categorical variables were compared between groups using the chi-square test. Receiver operating characteristic (ROC) curves were generated; the variable of interest was the NIHSS score at admission and the NIHSS score variation immediately after treatment, and the outcome variable was the mRS score at all possible cutoff points. The cutoff points of the NIHSS score or the NIHSS score variation that could best discriminate the mRS score were determined by the highest sum of sensitivity and specificity (best Youden index).

The association between the type of treatment and primary outcome was assessed using binary logistic regression, with the primary outcome as the dependent variable and the type of treatment as the variable of interest. Confounding variables were selected when *p* < 0.05 was reached among the different modalities of treatment. Then, an automatic backward stepwise variable exclusion method (excluding variables with *p* ≥ 0.05 at any step) was used to select variables for the final model. The significance level was set at *p* < 0.05, for all analyses. Data analysis was performed using IBM SPSS Statistics® Version 21 software.

## Results

**Figure 1:**
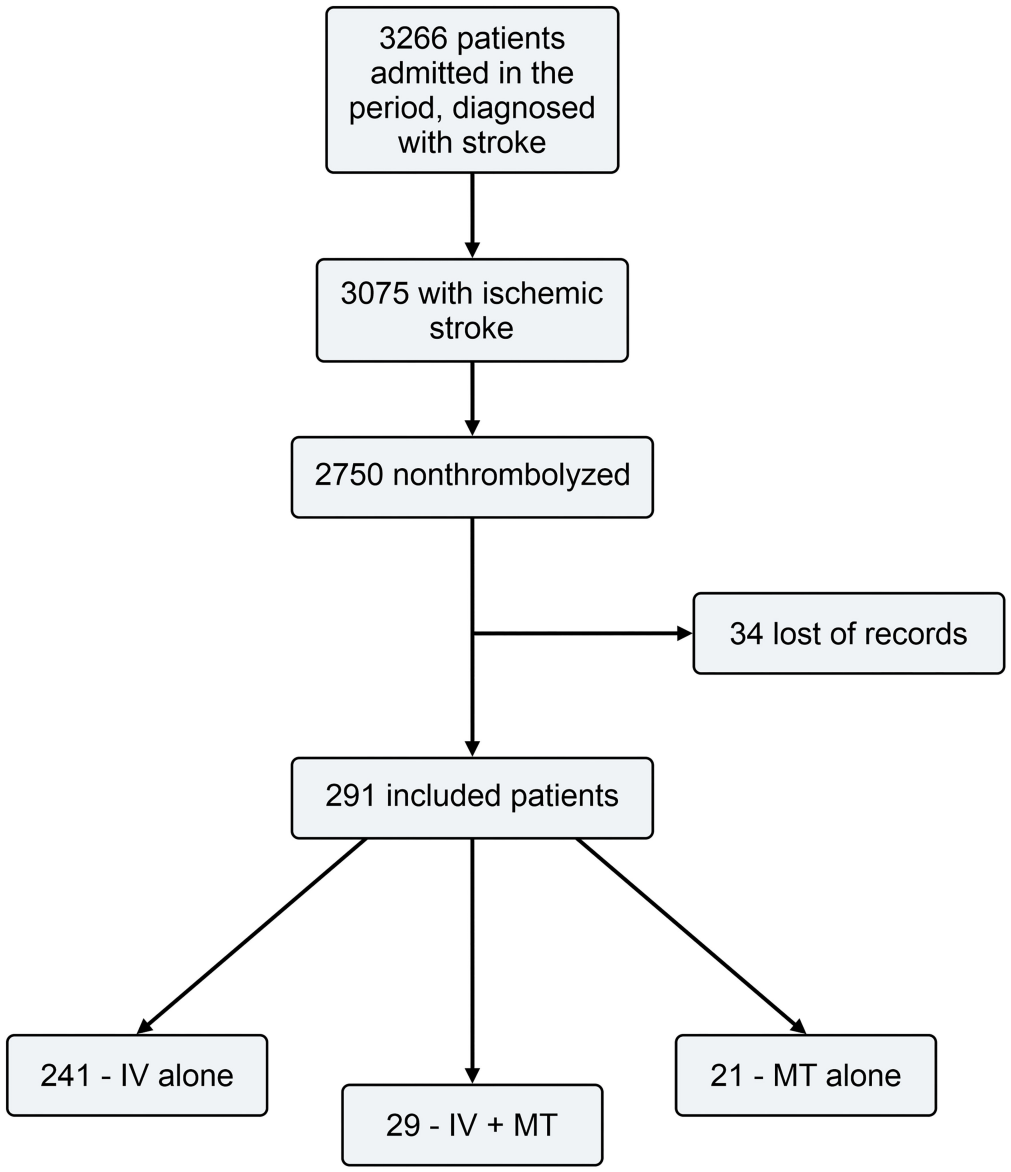
Flow chart.

A flowchart of the patients included in the study is shown in Figure 1. Of the 291 patients who underwent cerebral reperfusion therapy, 241 patients underwent only IVT, 21 underwent MT, and 29 underwent IVT with MT. The eligibility rate for cerebral reperfusion was 9.46%.

Table 1 shows the patients' demographic and clinical data according to the type of treatment received. In comparison to the MT alone and IVT with MT groups, IVT alone group had a higher proportion of elderly and Caucasian patients, as well as patients with lower post-treatment NIHSS scores, higher ASPECTS, shorter symptom-to-door and door-to-treatment time, and lower rate of symptomatic hemorrhage and surgical treatment. The IVT alone group also had a lower death rate and prevalence of disability at hospitalization and after 90 days of ictus. Successful recanalization (scores 2b and 3) immediately after thrombectomy was 76.2% in the MT group and 72.4% in the IVT with MT group.

**Table 1:** Demographic and clinical data according to the type of treatment.

	**IVT (*n* = 241)**	**MT** **(*n* = 21)**	**IVT + MT (*n* = 29)**	***P***
Age (years)	68 ± 12.8	61 ± 15.4	65 ± 12.9	0.037
Females (%)	133 (55.0)	10 (48.0)	21 (72.0)	0.171
White (%)	217 (90.0)	15 (71.0)	25 (86.0)	0.031
NIHSS at admission	12.4 ± 6.8	13.5 ± 7.3	14.4 ± 6.8	0.32
NIHSS after IVT	8.8 ± 6.6	-	17.8 ± 6.2	<0.001
Diabetes mellitus (%)	68 (28.0)	8 (38.0)	11 (38.0)	0.436
Hypertension (%)	185 (77.0)	15 (71.0)	20 (69.0)	0.616
Non-smoker (%)	123 (51.0)	9 (43.0)	17 (59.0)	0.604
SBP at admission (mm Hg)	159 ± 29.6	146 ± 40.5	153 ± 29.1	0.098
DBP at admission (mm Hg)	90 ± 16.4	83 ± 18.1	86 ± 19.3	0.134
Serum creatinine (mg/dl)	1.1 ± 0.7	0.9 ± 0.3	1.0 ± 0.3	0.259
ASPECTS	8.8 ± 1.3	7.3 ± 1.5	8.0 ± 1.8	<0.001
Symptom-to-door time (min)^†^	135 ± 90	218 ± 165	141 ± 105	0.001
Door-to-treatment time (min)^†^	61 ± 44	246 ± 171	222 ± 111	<0.001
ECASS (%)				
0	185 (76.8)	12 (57.1)	18 (62.1)	0.019
1	8 (3.32)	1 (4.8)	3 (10.3)	
2	14 (5.81)	3 (14.3)	0 (0.0)	
3	25 (10.4)	5 (23.8)	4 (13.8)	
4	9 (3.67)	0 (0.0)	4 (13.8)	
Recanalization rate (%)				
TICI 0	NA	2 (9.5)	2 (6.9)	
TICI 1	NA	2 (9.5)	4 (13.8)	
TICI 2a	NA	1 (4.8)	2 (6.9)	0.678
TICI 2b	NA	6 (28.6)	5 (17.2)	
TICI 3	NA	10 (47.6)	16 (55.2)	
Hemicraniectomy	5 (2.1)	3 (14.3)	6 (20.7)	<0.001
Death in hospitalization	29 (12.0)	2 (9.5)	11 (37.9)	0.001
Death at 90 days	36 (14.9)	4 (19.0)	14 (48.3)	<0.001
mRS > 2 at 90 days^‡^ (%)	35/203 (17)	7/17 (41)	6/15 (40)	0.014

*IVT, intravenous thrombolysis; MT, mechanical thrombectomy; NIHSS, National Institute of Health Stroke Scale; SBP, systolic blood pressure; DBP, diastolic blood pressure; ASPECTS, Alberta Stroke Program Early Computed Tomography Score; ECASS, European Cooperative Acute Stroke Study; TICI, Thrombolysis in Cerebral Infarction scale*.

† *non-parametric test*.

‡ *only survivors*.

The incidence of death within 90 days after correction for age, the ASPECTS, and the ECASS criteria was increased by five times in patients who underwent IVT with MT (OR: 5.192; 95% CI 2.069–13.027; *p* = 0.000; Table 2). In addition, the prevalence of disability within 90 days after correction for age, the ASPECTS, and the ECASS criteria was increased by three times in patients who underwent IVT with MT (OR: 3.530; 95% CI 1.376–9.055; *p* = 0.009; Table 3).

**Table 2:** Multiple logistic regression analysis evaluating the outcome of death at 90 days after discharge according to the three types of treatments.

**Variables**	**OR**	**CI 95%**		***P***	
Step 1	**IVT (Ref)**				
	MT	0.971	0.190	4.956	0.972
	IVT + MT	5.264	2.084	13.294	0.000
	Age (in years)	1.042	1.013	1.072	0.004
	Ictus-door time (in min)	1.001	0.996	1.006	0.740
	Door-treatment time (in min)	1.001	0.998	1.004	0.490
	SBP at admission	0.998	0.987	1.009	0.714
	NIHSS at admission	1.000	0.952	1.050	0.993
	ASPECTS	0.743	0.598	0.924	0.007
	**ECASS 0 (Ref)**				
	ECASS 1	0.585	0.087	3.929	0.581
	ECASS 2	1.275	0.317	5.138	0.732
	ECASS 3	1.552	0.606	3.979	0.360
	ECASS 4	6.266	1.738	22.592	0.005
Final model	**IVT (Ref)**				
	MT	1.276	0.364	4.469	0.703
	IVT + MT	5.192	2.069	13.027	0.000
	Age (in years)	1.039	1.011	1.067	0.006
	ASPECTS	0.747	0.603	0.925	0.007
	**ECASS 0 (Ref)**				
	ECASS 1	0.556	0.086	3.601	0.538
	ECASS 2	1.177	0.298	4.649	0.816
	ECASS 3	1.515	0.598	3.838	0.381
	ECASS 4	6.579	1.856	23.328	0.004

*OR, Odds ratio; CI, confidence interval; IVT, intravenous thrombolysis; MT, mechanical thrombectomy; NIHSS, National Institute of Health Stroke Scale; SBP, systolic blood pressure; DBP, diastolic blood pressure; ASPECTS, Alberta Stroke Program Early Computed Tomography Score; ECASS, European Cooperative Acute Stroke Study*.

**Table 3:** Multiple logistic regression analysis evaluating the outcome of moderate disability (modified Rankin scale score ≥ 3) 90 days after discharge according to the three types of treatments.

**Variables**	**OR**	**CI 95%**		***P***	
Step 1	IVT (Ref)				
	MT	1.548	0.431	5.558	0.503
	IVT + MT	3.515	1.367	9.039	0.009
	Age (in years)	1.035	1.014	1.058	0.001
	Ictus-door time (in min)	1.001	0.997	1.005	0.545
	Door-treatment time (in min)	1.000	0.997	1.003	0.949
	SBP at admission	1.000	0.991	1.008	0.925
	NIHSS at admission	0.998	0.961	1.037	0.913
	ASPECTS	0.763	0.632	0.921	0.005
	**ECASS 0 (Ref)**				
	ECASS 1	0.266	0.055	1.282	0.099
	ECASS 2	1.032	0.352	3.021	0.955
	ECASS 3	2.538	1.087	5.930	0.031
	ECASS 4	5.619	1.138	27.731	0.034
Final model	**IVT (Ref)**				
	MT	1.938	0.707	5.317	0.199
	IVT + MT	3.530	1.376	9.055	0.009
	Age (in years)	1.035	1.014	1.056	0.001
	ASPECTS	0.765	0.635	0.921	0.005
	**ECASS 0 (Ref)**				
	ECASS 1	0.263	0.056	1.247	0.093
	ECASS 2	1.034	0.358	2.991	0.951
	ECASS 3	2.549	1.104	5.884	0.028
	ECASS 4	5.427	1.126	26.154	0.035

*OR, Odds ratio; CI, confidence interval; IVT, intravenous thrombolysis; MT, mechanical thrombectomy; NIHSS, National Institute of Health Stroke Scale; SBP, systolic blood pressure; DBP, diastolic blood pressure; ASPECTS, Alberta Stroke Program Early Computed Tomography Score; ECASS, European Cooperative Acute Stroke Study*.

**Figure 2:**
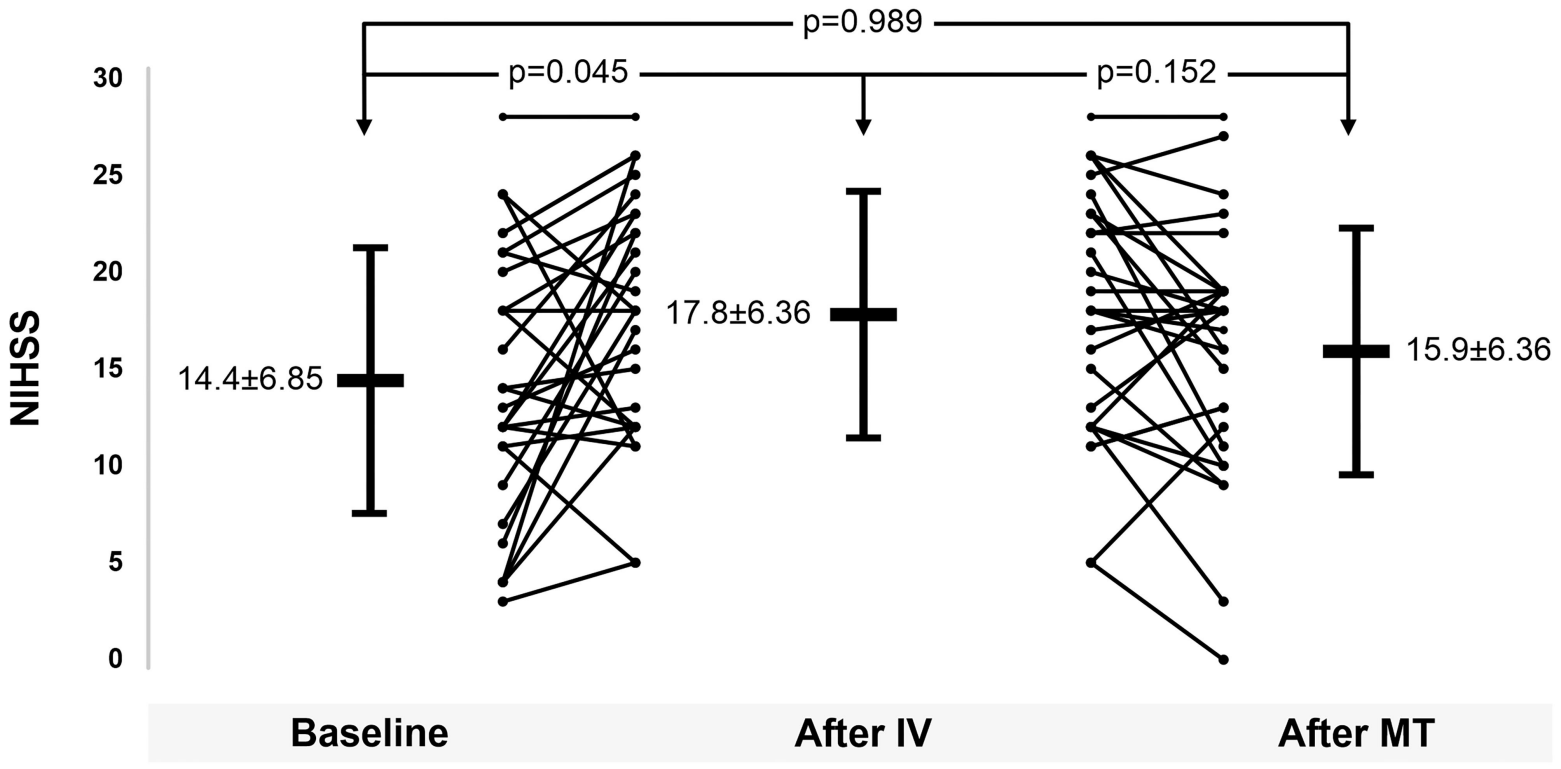
National Institutes of Health Stroke Scale score variation after treatments.

Figure 2 shows the variation in the NIHSS score in the IVT with MT group. The initial mean NIHSS score was 14.4 ± 6.85, but it increased to 17.8 ± 6.36 after the completion of IVT (*p* = 0.045) and then returned to 15.9 ± 6.36 (*p* = 0.152). There was no significant difference between the initial NIHSS score and that after MT (*p* = 0.989).

**Figure 3:**
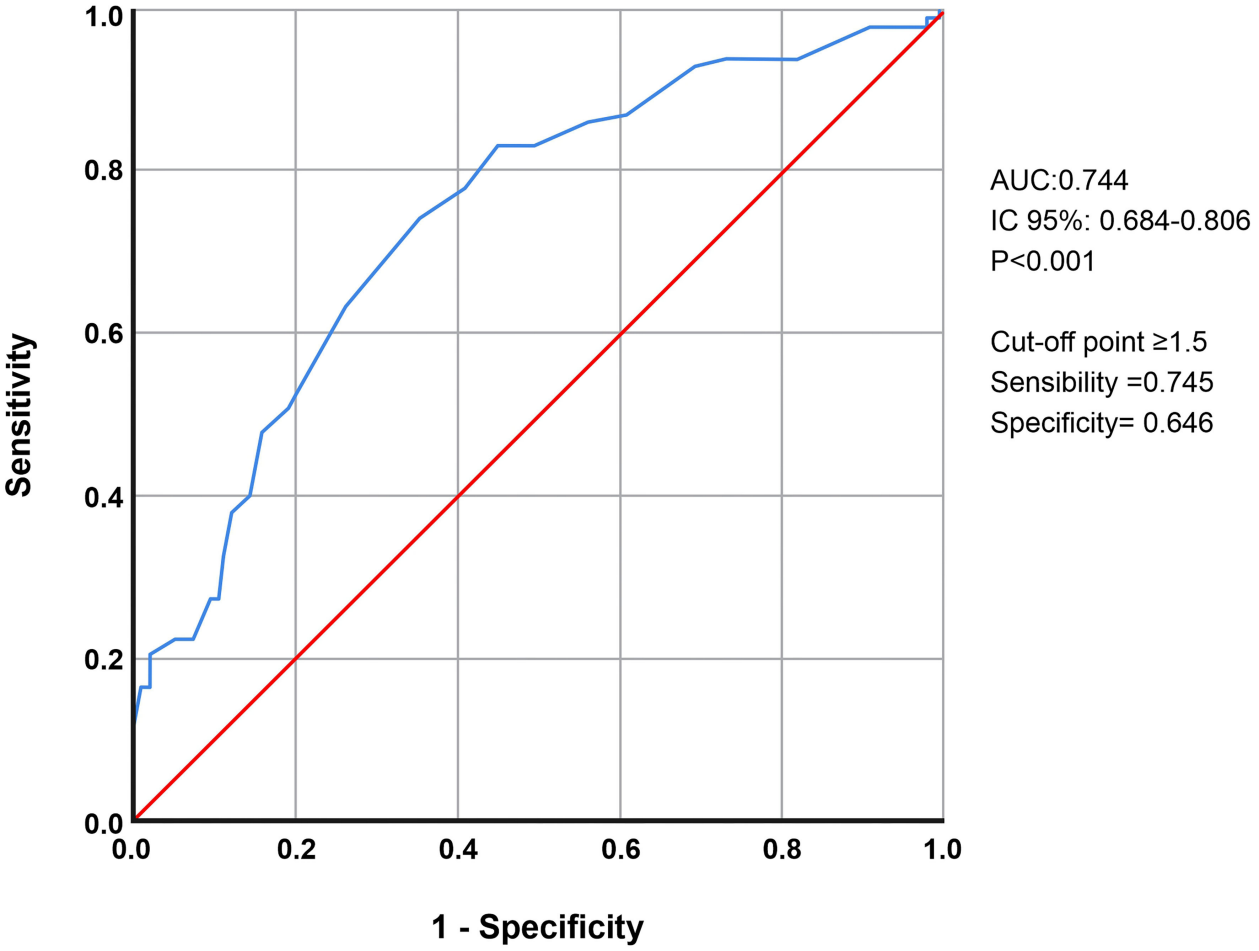
ROC curves of NIHSS scores after IVT and MT compared to the mRS scores.

Figure 3 shows the ROC curves of NIHSS scores after IVT and MT compared to the mRS scores. For patients in whom the NIHSS score increased or decreased by 2.5 points, the sensitivity was 0.74 and the specificity was 0.65, indicating severe disability or death in these patients (mRS score, 5 or 6).

## Discussion

The study demonstrated that IVT with MT increased the risk of death and disability in patients within 90 days after ictus compared with others groups (IVT and MT alone), therefore, there were differences in the clinical features at onset between the groups that received treatment with IV thrombolysis, mechanical thrombectomy, and finally those that received both treatments. A 2.5-point variation in the NIHSS score after the IVT in the IVT with MT group indicated worse outcomes with high sensitivity and specificity. However, regardless of the type of treatment, higher ASPECTS was a protective factor, and the presence of symptomatic hemorrhagic transformation increased the chance of death in patients who underwent MT alone.

Our study provides a detailed overview of treatment effectiveness, mortality rate, complications, and functional outcomes of stroke in LMICs. The IVT alone group had a lower NIHSS score, higher ASPECTS, lower rates of symptomatic hemorrhage and surgical treatment, and better outcomes at 90 days. Expanding access to alteplase therapy for acute ischemic stroke is a multifaceted approach. Specific considerations based on the region, population, and healthcare resources should be considered for each strategy. Neuroimaging approaches to identify alteplase-eligible patients are a recent development in acute stroke care that holds promise for increasing alteplase treatment rates (28).

Despite a higher NIHSS score in the MT alone group, the benefits of treatment were maintained at 90 days. Stroke severity after MT and rate of symptomatic hemorrhage were higher in the IVT with MT group. In a small Brazilian cohort, a higher rate of functional independence was observed in patients who received intra-arterial thrombolysis plus IVT than in those who received IVT alone (29). The DEVT randomized clinical trial showed that among patients with ischemic stroke who received treatment within 4.5 h from onset, MT alone met the prespecified statistical threshold for non-inferiority for 90-day functional independence outcome compared to IV alteplase followed by MT (30). Looking to the MT alone group, we noted better results, maybe because it was already too late for IVT (>4.5 h) or there was a contraindication for that and the conditions to do a MT were all available when indicating that treatment.

Access to catheters is limited in developing countries. The main drawback of MT in everyday practice is the irregular supply of catheters and materials. Moreover, devices for neurointerventional procedures are acquired through an auction process where cheaper devices are more easily acquired but may not be of the best quality (31). Despite these drawbacks, our study reported a higher rate of acceptability for cerebral reperfusion therapy than other studies in Latin America (32) because of the integration between stroke units and emergency prehospital services, as well as the availability of the semi-automatic stroke databank.

In our study, we observed that the majority of acute ischemic stroke patients who arrived early to the emergency department received IV thrombolysis treatment and had lower NIHSS scores. Patients who arrived late received treatment with MT and had severe neurological deficits. Severely ill and younger patients usually arrive early to the emergency department. The selection of patients for the stroke center can be via SAMU (direct access) or via the central regulation of the health system, which regulates municipalities with more than 50 km from the stroke center. However, the most serious cases that underwent thrombectomy, 60% or more were from municipalities far from Botucatu (comprehensive stroke center) and came through an alternative regulatory route. In the IVT group, most patients arrived faster due to being via SAMU from Botucatu or nearby municipalities (<50 km). This also reflects the need for better structuring and integration of the pre-hospital network across the entire range of the stroke center's area of operation.

Selection of patients who undergo MT after IVT should be careful and more rigorous. In LMICs, stroke is more severe due to several risk factors, including high tobacco usage (33, 34); a greater prevalence of diabetes mellitus, hypertension, cardiovascular diseases, dyslipidemia, and obesity (35); a lower prevalence of atrial fibrillation (36, 37); and a higher mortality rate (38). It is unclear whether current healthcare systems in most LMICs are well equipped to manage this enormous burden of stroke. The model of our service when the patients were attended was to perform IVT and waiting for clinical improvement, which may have generated a delay to indicate MT.

Recently, a randomized clinical trial (RESILIENT trial) was performed to evaluate the safety and efficacy of MT in the public health system of Brazil. The authors concluded that endovascular treatment within 8 h of onset of stroke symptoms in conjunction with standard care resulted in better functional outcomes at 90 days after stroke than standard care alone. In addition, substantial reperfusion in the thrombectomy group (≥50% of the affected territory) was achieved in 91 of the 111 patients (82.0%). However, it should be noted that this positive result of endovascular treatment was achieved due to the careful selection of patients for MT—patients with an ASPECTS of <6 on CT or <5 on MRI and complete absence of leptomeningeal collaterals on CT angiography. In addition, patients needed to have a baseline infarct volume of <70 ml, a ratio of volume of ischemic tissue to baseline infarct volume of ≥1.8 ml, and an absolute volume of potentially reversible ischemia (penumbra) of ≥15 ml (16).

There is a gap in stroke care in Brazilian public health; however, the RESILIENT study introduced expectations of better health services, cost-effectiveness, and improved care in LMICs. In the present study, substantial reperfusion (TICI 2b and 3) was achieved in 21 of the 29 patients (72.4%) in the IVT with MT group, and 16 of the 21 patients (76.2%) in the MT alone. The patients indicated for MT were considered according to the severity of stroke assessed using the NHISS; therefore, the individuals had worse neurological conditions. Based on these findings, we believe that other selection criteria should be considered for the indication of MT, such as ASPECTS, collateral circulation analysis, and CT perfusion to assess the ischemic core (39, 40).

Despite our results showing inferior outcomes among patients receiving IVT and MT, we believe that it occurred in our particular context, which reflects the difficulties of access to MT rather than a risk of the combined therapy. In the RESILIENT trial, the median time from stroke onset to treatment was 170 min, while in our cases, it took about 220-240 min. Therefore, even though our patients were not so different from the RESILIENT's at the baseline (regarding to age and NIHSS at admission) and our recanalization rates were close to the RESILIENT's, we had more patients with complications (symptomatic intracerebral hemorrhage and need for decompressive craniectomy), which may be linked to that difference on time to treatment. This difference points to the need for LMIC to expand the accessibility to MT; it is not enough to have MT available: it is needed to have it largely and promptly available in order to ensure its benefits (41).

Limitations of cerebral intervention include the high level of procedural skills required by the operator, technical limitations associated with catheter technology in middle income countries, lengthy duration of the method, and high costs (42, 43). However, consistent learning and experience performing approximately 30–50 radial interventions can improve the skills of interventionalists, enhancing their efficiency and level of comfort with this procedure (44, 45). Our center has a specialized interventional neuroradiologist with many years of experience; however, the intervention laboratory is located in a teaching hospital with the aim of teaching students and residents in neurology. Despite the success of reperfusion rates, we also had the technical limitation of lack of materials provided by the public health system, Angio suites shared with other specialties, reducing its immediate availability for stroke management, increasing the time for treatment and reducing the chance of better outcomes.

In addition, prehospital procedures can improve the efficacy of acute reperfusion therapy. System-based approaches have improved in-hospital temporal parameters, maximized the utility of reperfusion therapies, and improved clinical benefits to patients (46). Several studies on prehospital stroke workflow optimization (PSWO) have implemented various strategies and shown success in reducing workflow time delays and patient treatment rates (47). Chowdhury et al. showed that improved IVT triage is significantly associated with improved rates of IVT and thus can increase the number of potential IVT candidates. Furthermore, PSWOs were able to significantly reduce delays in several time metrics related to stroke workflow. Improved IVT triage, large vessel occlusion bypass, and mobile stroke units (MSUs) were all found to significantly reduce door-to-treatment time. Our center is connected to the urgent and emergency system (SAMU), and the stroke code can facilitate an early and organized therapeutic approach. Therefore, MSUs could be a decisive factor in reducing treatment time and could have long-term clinical benefits.

We acknowledge the limitations of our study. First, this was a retrospective study with a small cohort. Second, this study included a convenience sample, and there was a constant learning curve since this was a university hospital. Third, the number of patients treated with the thrombectomy modality was smaller than that of patients treated with other cerebral reperfusion modalities. Fourth, we did not use collateral and perfusion imaging information to improve patient selection. CT perfusion was not available in many periods of the retrospective study due to technical failures. In addition, this research was conducted in a middle-income country with all the limitations associated with that status.

The strengths of this study include its longitudinal design and the large sample size of the IVT group. Furthermore, all patients were assessed with a comprehensive neurological assessment. We believe that strategies to improve stroke care, which have been successfully implemented in high-income countries, may be useful for adaptation and implementation in LMIC settings to improve overall therapeutic outcomes. Our study does not emphasize the effectiveness of cerebral reperfusion therapies but emphasizes clinical predictors that might interfere with the neurological outcomes of individuals who have undergone such therapies.

## Conclusion

Altogether, a 2.5-point variation in NIHSS score after reperfusion in the IVT with MT is an indicator of worse outcomes. In our particular context, patients receiving the combination of IVT and MT had inferior results, which probably reflects challenges to optimize MT in LMIC.

We highlight the importance of optimizing treatment in the acute phase for better outcomes in stroke patients, especially for occlusion of large vessels. In addition, there should be better integration of the pre-hospital system with in-hospital management, associated with more established thrombectomy protocols.

## Data Availability Statement

The raw data supporting the conclusions of this article will be made available by the authors, without undue reservation.

## Ethics Statement

The studies involving human participants were reviewed and approved by Institutional Review Board at Botucatu Medical School. The Ethics Committee waived the requirement of written informed consent for participation.

## Author Contributions

EV, CF, LM, and RB: substantial contributions to the conception or design of the work. NEF, GL, PH, SZ, GM, NCF, LM, JS, and FW: acquisition, analysis, and interpretation of data for the work. NEF, GL, PH, LM, and RB: drafting and critical revision of the manuscript for important intellectual content. NEF, GL, PH, SZ, GM, NCF, LM, JS, FW, EV, CF, LM, and RB: final approval of the version to be published. All authors contributed to the article and approved the submitted version.

## Conflict of Interest

The authors declare that the research was conducted in the absence of any commercial or financial relationships that could be construed as a potential conflict of interest.

## Publisher's Note

All claims expressed in this article are solely those of the authors and do not necessarily represent those of their affiliated organizations, or those of the publisher, the editors and the reviewers. Any product that may be evaluated in this article, or claim that may be made by its manufacturer, is not guaranteed or endorsed by the publisher.
